# Optimization of Electrochemical Visualization of Latent Fingerprints with Poly(Neutral Red) on Brass Surfaces

**DOI:** 10.3390/polym13193220

**Published:** 2021-09-23

**Authors:** Gabriela Broncová, Tereza Slaninová, Miroslava Trchová, Vadim Prokopec, Pavel Matějka, Tatiana V. Shishkanova

**Affiliations:** 1 Department of Analytical Chemistry, University of Chemistry and Technology Prague, Technická 5, 166 28 Prague 6, Czech Republic; ter.slaninova@seznam.cz (T.S.); Vadim.Prokopec@vscht.cz (V.P.); Tatiana.Shishkanova@vscht.cz (T.V.S.); 2 Central Laboratory, University of Chemistry and Technology in Prague, Technická 5, 166 28 Prague 6, Czech Republic; Miroslava.Trchova@vscht.cz; 3 Department of Physical Chemistry, University of Chemistry and Technology Prague, Technická 5, 166 28 Prague 6, Czech Republic; Pavel.Matejka@vscht.cz

**Keywords:** latent fingerprints, brass cartridge, electrochemical deposition, poly(neutral red), visualization

## Abstract

This study is focused on the visualization of latent fingerprints on brass surfaces using the method of electrochemical deposition of a polymer film based on poly(neutral red) (PNR). The experiment included (i) optimization of conditions of electrochemical deposition of PNR on brass surfaces, (ii) ATR-FTIR spectroscopic characterization of PNR-modified substrates, and (iii) identification of characteristic details on visualized fingerprints on fired brass cartridges. For electrochemical visualization, it is necessary to keep in mind both kind and “story” substrates. Experimental findings showed that electrochemical visualization carried out on brass plates is a step forward before known findings described in the literature and gives simultaneously a new approach for criminalists in the fight against crime.

## 1. Introduction

Fingerprints are currently one of the most widely used means of forensic identification. Fingerprint identification is based on the presence of features that correspond to the details of the second level of papillary lines [1,2,3]. Fingerprints can also be examined at the first and third levels of detail; however, they cannot be used for unambiguous identification, but only to exclude the perpetrator [4,5,6].

In contemporary forensics, several techniques are used to visualize latent fingerprints; they are generally divided into optical, physical, physico-chemical, and chemical methods [7,8,9]. In addition to conventional techniques (powdering, cyanoacrylate vapors, ninhydrin), some newer and more sophisticated methods are used and described in the studies. The choice of a suitable technique depends mainly on the nature of the surface on which the fingerprint is to be made visible/visualized [9].

Non-destructive optical visualization techniques use the optical properties of fingerprints [1,9,10]. Unlike other techniques, optical methods also provide information about the molecular structure of the fingerprint. This group includes methods based on infrared absorption, Raman scattering, UV absorption, etc. [9]. Physical techniques are used to visualize the adhesive properties of fingerprints, where the specific reagents adhere to the greasy components of the fingerprint [7,10]. Examples of physical methods are pulverization, Kelvin scanning probing, or vacuum metal deposition [9]. When physico-chemical visualization methods are used, the chemical agent is bound to or dissolved in the area of the applied fingerprint [1]. This group of methods includes cyanoacrylate vapors, iodine vapors, multiple metal deposition, electrochromic film deposition, and others [9]. The last group contains chemical methods that use direct reactions of a chemical agent with fingerprint components to form a colored product [1,2,7]. These commonly used reagents are mainly ninhydrin, silver nitrate, or Gun blue solution [9].

From the point of view of forensics, the most important step is the visualization of latent prints on metal surfaces, which are commonly used in criminal activities (weapons, cartridges, knives) [4,6,9,11]. However, the possibility of visualizing fingerprints on cartridge cases is of the greatest importance. There are only a few techniques that can successfully make such fingerprints visible [9,12,13].

**Figure 1:**
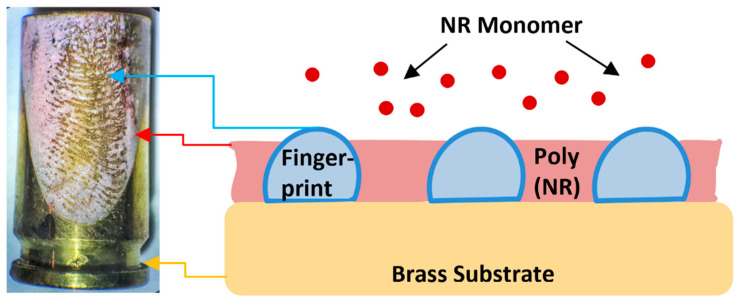
Scheme of visualization of fingerprint on brass cartridge using poly(neutral red) film.

This work is in part focused on the visualization of fingerprints electrochemically using polymer films. This is a relatively new technique, which has been studied in recent years with the possibility of use in dactyloscopy [4,12,14]. The principle of the method is the application of a conductive polymer on the desired surface containing the fingerprint (rich in lipid components), which gradually develops a negative image of the fingerprint (see Figure 1), because the presence of fatty acids in the latent fingerprint acts as a non-conductive mask, preventing the occurrence of the electrochemical process [6,10,12,15]. The polymer film is applied only in the background outside the fingerprint area [4,6,9,10]. The thickness of the applied film should always be lower compared to the fingerprint [4,6], since thicker films provide lower quality and clarity of the visible fingerprint [10]. After applying the film, the imprinted sample is placed in a second solution without the presence of the monomer, which subsequently changes the color of the polymer when the applied potential changes, resulting in contrast and thus better visibility, when the resulting imprint (outline) can be seen with the naked eye [4,9,10,12]. In general, some crucial properties of the dactyloscopic traces in terms of their visualization can be changed by tuning the ambient conditions (applied potential, pH, etc.) The great advantage of this method is the ability to make prints visible on a wide range of metal substrates (bronze, brass, lead, copper, nickel). These are mainly metals that are commonly used in the manufacture of cartridges [9].

The electrochemical deposition of conductive polymers appears to be complementary to conventional methods commonly used in practice nowadays [4,6,9,12], as they allow the examination of latent high-resolution fingerprints of second-level details used in the identification [1,4,6]. In some cases, it is possible to register even finer details of the third level [4,6]. The most widely used polymers in this field of research include polypyrrole (PPy) [8,12,14,15], polyaniline (PANI) [4,6,9,10], and polyethylenedioxythiophene (PEDOT) [4,6,9,12], which are effective in the visualization on both old and fresh prints [9]. Some conductive polymers are polychrome, which means that they occur in several color forms based on the oxidation state [6,16].

A lesser known polymer is poly(neutral red) (PNR) [17], which appears to be a promising novel material in this area [18]. The amino group NR located on the heteroaromatic phenazine ring facilitates the electropolymerization process. Despite the fact that there is no agreement on the mode of growth of these polymers, it is commonly believed that the mechanism begins with the formation of a radical cation from monomeric species [17,19]. Thus, the only way to obtain these polymers from monomer solutions is to apply cyclic voltammetry using the potentials at which radical species are formed. In addition, cyclic voltammetry is the preferred electrochemical technique for monitoring regular polymer growth [17,19].

Due to the high electrochemical activity of the phenazine monomer and polymers, they are used as sensitive layers or redox mediators with good electron transfer properties [19]. The mechanism of electrochemical polymerization, including the structure of the polymer, has been analyzed and discussed in previous studies [17,20]. Our primary goal in this study was to find a new application. The NR-based polymer film combines a number of properties that are important for forensic applications. These advantages include good adsorption on various metal surfaces [19,20,21], high mechanical strength [21], biocompatibility, long-term stability [19], and selectivity [17] on components/species specific to individuals and useful for their identification.

The rate of successful visualization of fingerprints on cartridges [9,12,13] is low in common cases [13,22]. Beresford et al. are the only ones who have investigated the visualization of latent prints on brass cartridges using electrochromic conductive polymers, specifically PANI [23]. The performed experiments compare visualization on unfired and fired cartridges. In the case of unfired cartridges, relatively high-quality images of papillary lines were obtained, but in the case of fired cartridges, only partial prints were visualized [23].

The aim of this work was to make fingerprints visible on various metal substrates (plates, cartridges) made of brass (Figure 1) due to the large application in the field of forensics, surface characterization, and optimization of the visualization method. In our preliminary studies, fingerprint visualization was successfully tested using electrochemically prepared polymer films (PNR films) on a platinum surface and with possibility of fingerprint visualization on brass [18]. The main goal of this work was to find the optimal conditions for the visualization of fingerprints using PNR films and analytical characterization on a brass plate, and the results were subsequently used to visualize fingerprints on fired brass cartridges.

It is essential that this possibility of using a simple method of visualizing fingerprints on fired cartridges based on the application of a suitable polymerized layer offer application in the future in the investigation of crimes.

## 2. Materials and Methods

### 2.1. Chemicals

For electrochemical cleaning of the cell and auxiliary Pt electrode, 0.5 mol∙L^−1^ of sulfuric acid (H_2_SO_4_, Penta, CR, 96% p.a.) was prepared. Acetone (Penta, CR) and ethanol (ethyl alcohol, Penta, CR, 96% p.a.) were used to clean prepared brass plates and fired brass cartridges, 0.1 mol∙L^−1^ of sodium nitrate (NaNO_3_, Lachema Brno, CR) was used as the supporting electrolyte, 0.002 mol∙L^−1^ of neutral red (NR, Lachema Brno, CR) was the substance applied for polymerization/visualization. A solution of 0.1 mol∙L^−1^ of potassium nitrate (KNO_3_, Lachema Brno, CR), 0.005 mol∙L^−1^ of potassium hexacyanoferrate (K_4_[(Fe(CN)_6_], Lachema Brno, CR), and 0.005 mol∙L^−1^ of potassium ferrocyanide (K_3_[Fe(CN)_6_], Lachema Brno, CR) was prepared for the purpose of electrochemical characterization experiments. All chemical reagents were of analytical grade, obtained from commercial suppliers, and used without further purification.

### 2.2. Instrumentation

Electrochemical experiments were performed using an Autolab PGSTAT 12 potentiostat/galvanostat (Eco-Chemie, Utrecht, The Netherlands) in a three-electrode cell, which included a reference argent chloride electrode (Ag/AgCl in 3M KCl), an auxiliary large-area Pt electrode, and a working electrode. For the purpose of electrochemical cleaning, a Pt wire electrode (diameter 0.4 mm, length 7 mm, UCT Prague) was used as the working electrode. For visualization of fingerprints, cut brass plates (size 15 mm × 25 mm) or fired brass cartridges (height 19 mm, diameter 9 mm, thickness 0.5 mm) were used as working electrodes.

A mobile phone (iPhone 7), a Leica DM2700 microscope connected to a Raman microscope, a Nikon eclipse LV100 light microscope with a ProgRes CT3 digital camera (5× objective, 10× eyepiece, 50× total magnification), a binocular magnifier BA 128 LED (1×/3× lens, 10× eyepiece-10×/30× total magnification), and a Nikon SMZ1500 stereoscopic zoom microscope were used for image acquisition and characterization. FTIR spectra were obtained using a Thermo Nicolet 6700 FTIR spectrometer equipped with a GladiATR attachment (attenuated total reflectance (ATR) technique).

### 2.3. Experimental Procedures

#### 2.3.1. Electrochemical Cell Cleaning

Prior to each experiment, the cell and auxiliary Pt electrode had to be electrochemically cleaned. Briefly, 10 mL of 0.5 mol∙L^−1^ of H_2_SO_4_ was pipetted into the electrochemical cell, and CV was performed in the potential range from −300 to 1800 mV (vs. Ag/AgCl). A total of 5 cycles at a scan rate (SR) of 100 mV∙s^−1^ were performed.

#### 2.3.2. Treatment of Plates and Cartridges Prior to the Application of Fingerprints

The metal plates were subsequently rinsed with distilled water, acetone, hot soapy water, ethanol, and air-dried before each experiment.

The preparation and application of fingerprints were performed according to the procedure described in a study by Beresford et al. [12]. Before applying the fingerprints to metal surfaces, the hands were washed with hot soapy water and then dried with a paper towel. The fingertips were rubbed against the forehead first, and then the two hands were rubbed together. Sufficiently visually greasy fingerprints were then applied to the brass surfaces.

#### 2.3.3. Fingerprint Visualization

The supporting electrolyte for visibility of the fingerprints on the brass plate or cartridge was 15 mL of 0.1 mol∙L^−1^ of NaNO_3_, into which 0.0076 g of neutral red (0.002 mol∙L^−1^) was strewed. The NR was dissolved using an ultrasonic bath. CV in the potential range from −300 to 600 mV (SR 50 mV∙s^−1^, 6 cycles) was used to visualize the fingerprints while stirring with a magnetic stirrer.

#### 2.3.4. Characterization of Properties of Brass Surfaces

Characterization of the metal surface properties was performed microscopically, electrochemically, and spectroscopically. Various types of microscopes were used (see Section 2.2, “Instrumentation”), which enabled us to obtain different types of images and focus on the characteristic markers of the fingerprints.

Spectroscopic characterization was performed at the central laboratories of UCT Prague by the collection of infrared spectra using the ATR-FTIR technique from a circular area with a diameter of 1–2 mm^2^ (macro-ATR technique). Spectra were recorded for (1) a brass plate containing the fingerprints (in further text called B-FP), (2) a brass plate with the PNR polymer coating (further called B-PNR), and (3) a brass plate with the fingerprints and the PNR film (further called B-FP-PNR). A powder sample of the NR monomer was also measured as a spectral reference.

Electrochemical characterization of the metal surfaces was performed in a solution of 0.1 mol∙L^−1^ of KNO_3_ in the presence of (Fe (CN)_6_)^4−^ and (Fe (CN)_6_)^3−^ ions (0.005 mol∙L^−1^). A potential range from −350 to 450 mV per cycle and varying scan rates— of 5, 10, 25, 50, 75, 100, and 150 mV∙s^−1^—were used. To characterize the brass surfaces, these experiments were performed for (1) a plate alone, (2) a plate containing fingerprints on both sides, (3) a plate with the PNR polymer on both sides, and (4) a plate with the fingerprints covered with a PNR film on both sides.

#### 2.3.5. Optimization of the Fingerprint Visualization Process

During the optimization of the fingerprint visualization process, it was necessary to find ideal conditions under which the most visible fingerprint is obtained and, at the same time, it is possible to repeat the visualization. The basic parameters that influenced the visualization process included (i) the selection of the supporting electrolyte, (ii) the monomer concentration, (iii) the potential cyclization ranges used, and (iv) the number of cycles. During the experiments, these parameters were systematically changed and optimized.

## 3. Results and Discussion

### 3.1. Electrochemical Visualization of Fingerprints by Deposition of Poly(Neutral Red) Film onto Brass Substrates

Our strategy for the electrochemical visualization of fingerprints was based on the following assumptions. The fatty acids that are present in fingerprints act as a mask, preventing the movement of electrons. Then, latent fingerprints on brass substrates are visualized through the deposition of a polymer film derived from PNR. Thus, the polymeric film is formed and deposited only in places where there is no imprinted mask, i.e., on the brass surface itself. The result is obtained as a negative fingerprint image [16,24]. A dynamic electrochemical method of poly(neutral red) coating preparation is a promising and effective approach in this area. This electropolymerization offers a number of advantages: (i) control of the polymerization process, (ii) thin-film synthesis, (iii) simplicity of polymer preparation, and (iv) good adhesion of the polymer to metal surfaces. The advantage of applying poly(neutral red) to various surfaces is also its distinctive color.

**Figure 2:**
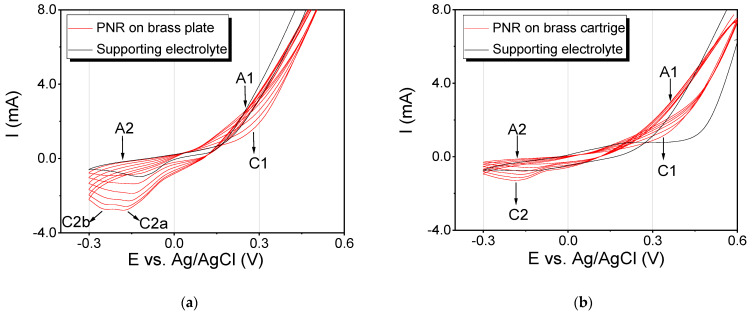
Cyclic voltammogram of the deposition of a PNR layer on the applied fingerprint on (**a**) a brass plate and on (**b**) a brass cartridge. Black curve, supporting electrolyte (0.1 mol∙L^−1^ NaNO_3_); red curve, polymerization of 0.002 mol∙L^−1^ of NR. The potential range of polymerization was from −300 to 600 mV (SR 50 mV∙s^−1^, 6 cycles).

The first part of the experiments was focused on the electrochemical deposition of the PNR film on two types of brass surfaces, namely plate and cartridge (Figure 2). In the case of the brass plate, two anodic peaks A1 and A2 and two cathodic peaks C1 and C2 were visible in the recorded voltammograms (Figure 2a). For the first anodic peak, A1 (250 mV), a significant increase in the current intensity and a shift of the potential to more positive values were observed. For the second anodic peak, A2 (−150 mV), the current intensity slightly decreased. In the cathodic range, two peaks were observed and marked as C2a (−125 mV) and C2b (−247 mV). We can point out that a gradual increase in the number of cycles results in a decrease in the peak current and its shift to more negative values. In the last cycle, it reached values of −179 mV. It has to be noted that from the fourth cycle, another cathodic peak C2b began to appear at −247 mV, while the current decreased again and the potential shifted to more negative values. This peak is probably related to the properties of the supporting material, which is composed mainly of copper, and oxides of this metal are naturally formed on the surface of the substrate [25]. The image of the visualized fingerprint made onto two identical brass plates is presented in the Supplementary Materials (Figure S1). Obviously, the thicknesses of the deposited PNR films are different on the two brass plates (compare Supplementary Materials Figure S1a,b). We assume that the main reason for this behavior is the difference in the thickness of the deposited PNR film, which is affected significantly by the different pressure applied to the fingerprint itself. Nevertheless, papillary lines were clearly visible in both images, and their more detailed examination will be discussed further (see Section 3.3, “Microscopical Visualization”).

In the case of brass cartridges, the cyclic voltammograms were similar to the ones obtained for the brass plates (Figure 2b): two anodic peaks (A1 = 350 mV and A2 = −180 mV) as well as two cathodic peaks (C1 = 335 mV and C2 = −183 mV) were observed. The only difference was in the current intensity, which can be explained by the surface size and shape variations.

### 3.2. Optimization of Electrochemical Visualization of Fingerprints

Another important step in this work was to optimize the parameters in the process of electrochemical fingerprint visualization (type of supporting electrolyte, monomer concentration, potential range, number of cycles). The main task was to obtain qualitative, repeatable, and sufficiently visible fingerprints.

In the case of brass as a substrate material, the choice of supporting electrolyte was clear in advance. Taking into account the chemical properties of brass, it was necessary to replace the acidic supporting electrolyte with neutral ones and thus to eliminate the chemical dissolving of the brass substrate. For this purpose, the following three types of electrolytes were tested: phosphate buffer, a mixture of phosphate buffer and NaNO_3_, and NaNO_3_. Based on obtained results, 0.1 mol∙L^−1^ of NaNO_3_ was selected as the optimal one. This supporting electrolyte was chosen due to the previously observed catalytic effect of NO_3_^−^ anions on the electropolymerization of neutral red [19].

The NR monomer concentration varied in the range from 0.005 to 0.002 mol∙L^−1^, and the ideal thickness of the polymeric film that visualized the fingerprint without its overlapping was attained for 0.002 mol∙L^−1^ of the NR monomer in the polymerization mixture.

**Figure 3:**
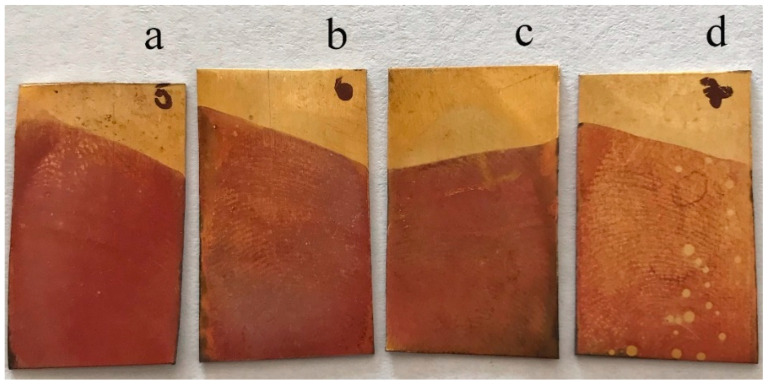
Optimization of the number of PNR deposition cycles for brass fingerprint visualization: (**a**) 10 cycles, (**b**) 8 cycles, (**c**) 9 cycles, and (**d**) 6 cycles.

In the initial phase, we applied wide potential ranges from −200 to 1000 mV, which did not lead to polymeric film formation at all. However, by gradually reducing the range, optimal conditions were found in the range from −300 to 600 mV. As can be seen, the gradual reduction from 10 up to 6 cycles is the most appropriate choice to obtain the optimal polymeric film for our purposes (Figure 3).

The exact film thickness was not measured; however, it was controlled by the number of cycles when applying the poly(neutral red) film to optimize the electrochemical visualization of the fingerprints. The optimal number was 6 cycles for sufficient visualization of fingerprints on brass using a PNR film. The thickness of the film should not exceed the thickness of the fingerprint. However, the thickness of the rest of the fingerprint can be as much as 0.1 μm, with considerable variability due to various factors, including moisture at the fingertip, applied pressure, and substrate condition [26]. A poly(neutral red) film of suitable thickness adheres only to the metal substrate and not to the fingerprint itself.

### 3.3. Microscopic Visualization

**Figure 4:**
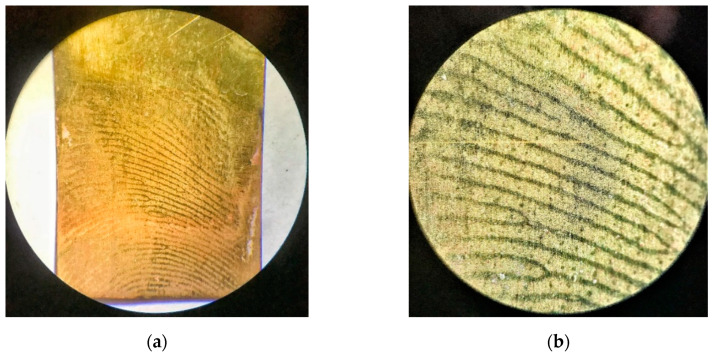
Visualized fingerprint on brass plates. Images were taken with a binocular magnifier: (**a**) 10× magnification and (**b**) 30× magnification.

**Figure 5:**
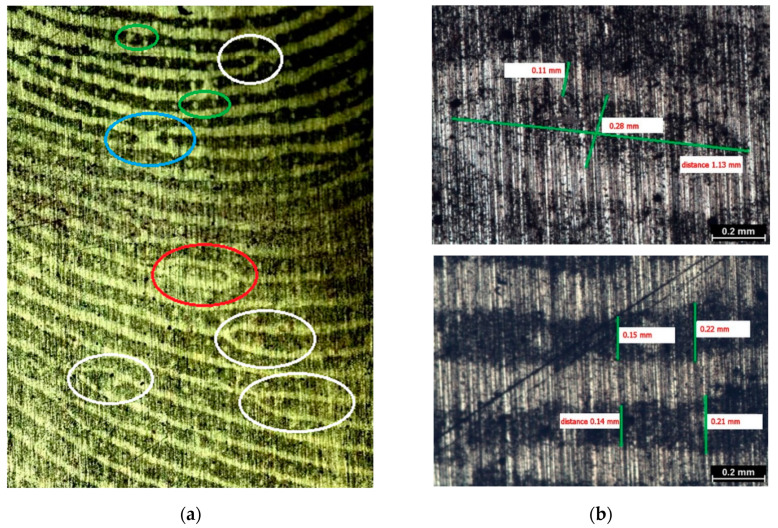
Image of details of the visualized fingerprint on the brass plate (**a**) obtained with a Leica microscope. Marked details: second level (markers): bifurcation (marked in white), eyelet (marked in red), and cross (marked in blue); third level: pores (marked in green). Image of details of the visualized fingerprint on the brass plate (**b**) taken with a Nikon eclipse light microscope. Characterization: top right, eyelet (marker) dimensions; bottom right, distance between papillary lines.

Visual characterization was performed using several different optical microscopes; the obtained images are presented in Figure 4 and Figure 5 and Supplementary Materials Figure S2. Details of the papillary fingerprint lines can be characterized on three different levels in terms of their shape and path [3,4,5,22]. Primarily, the quality and clarity of a fingerprint affect the structure of levels [6]. The first level is more general and does not serve for the identification of the person. The details of the second level (markers) are characteristic for each individual, so they are crucial in terms of the identification. Details of the third level, which include, e.g., pores, may be useful in the case when only partial fingerprints are available [4,5,6].

The second level is the most important and describes the path of the ridges of the papillary lines, as can be seen in Figure 4. The papillary lines differ significantly from each other, especially in their different course. Their placement and morphology in the fingerprint can be a key to distinguishing an individual (Figure 4b). Upon careful examination of the imprint, it can be observed that the patterns are usually not continuous (Figure 4a,b), with the exception of the eyelets (Figure 4a). In Figure 4b, these clear patterns are enlarged; they suddenly split, and there are several bifurcations of different lengths [5]. For a given dactyloscopic trace to be recognized as evidence leading to identification, it is necessary that it contain at least 10 markers [2].

Figure 5a shows, in particular, the characteristic details of the second level (markers), which serve for identification. Figure 5b shows the individual distances in the characteristic feature of the eyelet (see Figure 5b, top right) and between the papillary lines (see Figure 5b, bottom right). It can be seen from the bottom image of Figure 5b that the individual lengths of the papillary lines were similar in several places. The distance between the individual papillary lines at this point of the fingerprint was about 0.2 mm. Such information could also help in the case of individual identification.

### 3.4. Comparison of the Properties of a Brass Substrate Prior to and after Applying PNR to a Fingerprint

Furthermore, the properties of the brass surface were tested spectroscopically and electrochemically before and after applying a PNR film to the individual fingerprint in order to confirm the presence of individual layers (substrate, fingerprint, PNR film) and perform their subsequent characterization.

#### 3.4.1. Spectroscopic Characterization

Spectroscopic characterization was based on the collection of infrared spectra using the ATR technique, as it is an ideal non-destructive technique for the characterization of latent fingerprints in terms of their chemical composition [9,27,28]. Spectroscopic data can provide information not only regarding the chemical structure but at some point also in terms of the affinity/adhesion of the formed layers to the substrate surface. The components of latent fingerprints are mixtures of both water-soluble (hydrophilic) and water-insoluble (hydrophobic) substances derived from the eccrine and sweat glands of the skin [29]. Hundreds of endogenous inorganic (water, metal ions) and organic (proteins, amino acids, lipids) substances can be found in a fingerprint. However, some exogenous environmental contaminants, which enter the fingerprints when using cosmetics, using pharmaceuticals, handling food, etc., may also be present in trace amounts [3,30,31]. These can include various illicit drugs and explosive residues, which may later be of great importance in criminal investigations [31].

All test subjects had to wash their hands thoroughly before fingerprint samples were taken. For this reason, it was assumed that any remnants of skin or materials that the individual came in contact with before sampling could not contaminate the sample. After the hands were washed with soap and water, only the eccrine component of latent fingerprints was expected to predominate.

**Figure 6:**
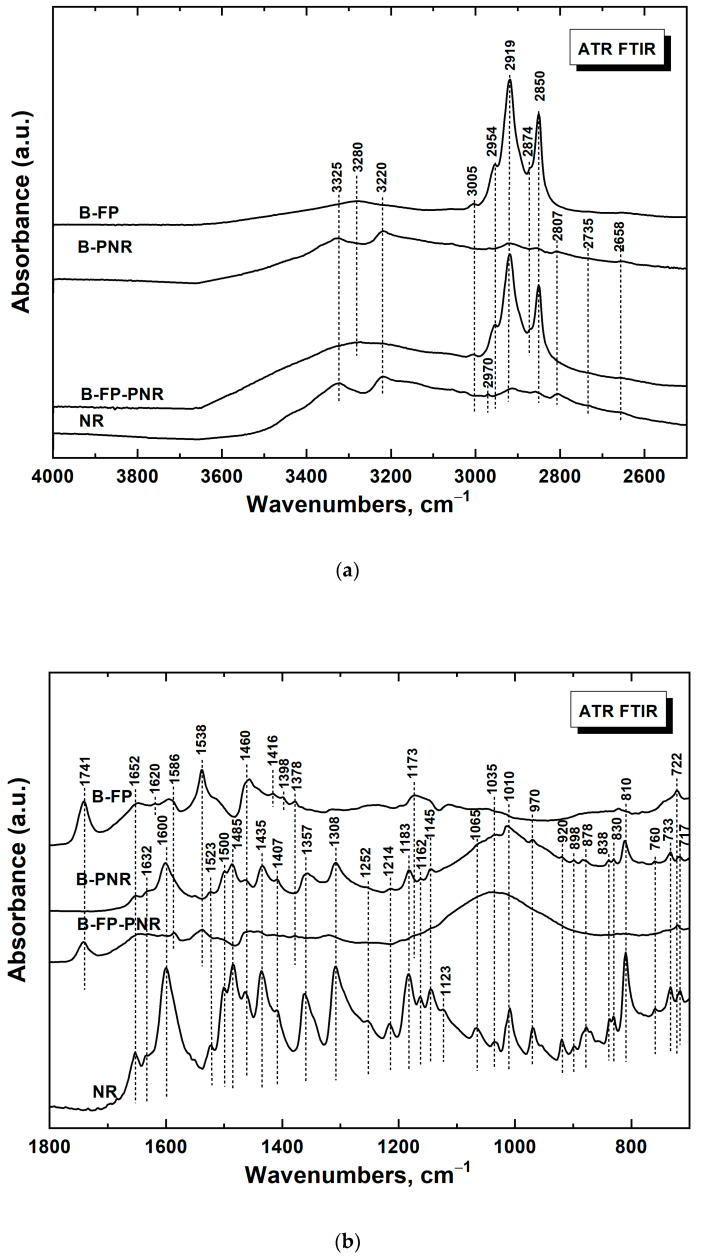
Infrared spectra of neutral red (NR) and the fingerprint and PNR film on the brass plate (B) in the wavelength range from (**a**) 2500 to 4000 cm^−1^ and (**b**) 1800 to 700 cm^−1^. B-FP, brass-fingerprint; B-PNR, brass-PNR film; B-FP-PNR, brass-fingerprint-PNR film.

To estimate the significance of our proposed visualization approach, the following series of experiments were focused on molecular spectroscopic characterization of four types of samples divided into two groups (based on the presence/absence of the fingerprint): (i) a brass plate with a deposited fingerprint in the absence (sample 1) and presence of a PNR film (sample 2) and (ii) a brass plate coated by a PNR film (sample 3) and powder NR (sample 4) as a standard/reference. Interpretation of the obtained spectra was performed according to the results reported in publications [29,32,33,34,35]. Figure 6a,b shows comparisons of the FTIR spectra of all samples studied on the brass surface supplemented with the reference powdered NR data in the range of 4000–2500 cm^−1^ and 1800–700 cm^−1^, respectively.

First, we identified the characteristic bands of human fingerprints/fingermarks (FP in the abbreviations) (sample 1). In the region from 2500 to 4000 cm^−1^ of the spectrum of fingermarks deposited on the brass support (sample 1, spectrum brass-fingerprint (B-FP); Figure 6a), bands typical of eccrine were detected. The bands with maxima at 2954 and 2919 cm^−1^ correspond to CH_3_ stretching vibrations, and the dominant bands at 2874 and 2850 cm^−1^ assigned to the CH_2_ stretching vibrations are derived from saturated aliphatic hydrocarbon chains, including long chains in sebaceous material. The relatively weak band at ca. 3005 cm^−1^ attributed to =CH stretching vibrations indicates the presence of unsaturated fatty acids in glycerides and wax esters of sebaceous components. The broad band with a maximum at ca. 3280 cm^−1^ corresponds to the hydrogen-bonded O–H stretching vibrations (often observed for human sweat and human/pig skin) [29,35].

In the region below 1800 cm^−1^ (sample 1, spectrum B-FP; Figure 6b), the band at 1741 cm^−1^ is assigned to the C=O stretching vibrations of saturated esters from the sebaceous component. The bands with maxima at 1652 and 1538 cm^−1^ belong most probably to the amide I (C=O stretching) and amide II (N–H deformation) of proteins/keratins and ceramides contained in skin debris [35]. The other bands in the region from 1700 to 1540 cm^−1^ (1652 and 1586 cm^−1^) are assigned to the O–H or N–H bending vibrations of water, lactic acid, or urea, which are present at the surface of human skin and in sweat. The bands with maxima at 1460 and 1378 cm^−1^ belong to CH_2_ and CH_3_ symmetric bending vibrations, respectively. The band with a maximum at 1173 cm^−1^ corresponds to CH_2_ twisting-and-rocking coupled vibrations. The peak observed at 722 cm^−1^ belongs to CH_2_ rocking vibrations on saturated aliphatic chains [29].

Second, the bands of the NR skeleton were identified in the spectrum of sample 3 (PNR on brass, spectrum brass-PNR (B-PNR); Figure 6a) using the reference spectrum of sample 4, the powdered NR (Table 1). It should be stressed that the absorption of the deposited PNR was low because of the low amount of materials deposited on the brass support. Nevertheless, almost all bands of the NR skeleton observed for the monomer were found in the spectrum of PNR. The only, nevertheless crucial, difference between the spectrum of B-PNR (sample 3) and NR powder (sample 4) was the presence of a broad band from 1200 to 900 cm^−1^, with a maximum at about 1035 cm^−1^ in the case of the B-PNR spectrum, which is empirically attributed to the stretching vibrations of newly formed C–N bonds in PNR, revealing the polymerization of NR units on the brass surface.

Finally, the bands of both FP chemical components and PNR were evaluated by comparing the spectra of human fingerprints deposited on brass and covered with PNR (sample 2) with the spectra of samples 1 and 3. In the region from 4000 to 2500 cm^−1^, the spectrum of sample 2 (spectrum brass-fingerprint-PNR B-FP-PNR) in Figure 6a) corresponds mainly to the spectrum of sample 1, where the human fingermark was deposited on pure brass (spectrum B-FP in Figure 6a). The quite weak bands of PNR (two rather broad bands with maxima at 3325 and 3220 cm^−1^ and weak features below 3000 cm^−1^ in the spectrum of sample 3) were overlapped by the evidently stronger bands attributed to human sweat and skin debris. In the region below 1800 cm^−1^ (spectrum B-FP-PNR in Figure 6b), we observed the same bands as in the case of the fingerprints on pure brass (sample 1, spectrum B-FP in Figure 6b) as well as the broad band with a maximum at 1035 cm^−1^ observed in the spectrum of PNR deposited on the brass support (sample 3, spectrum B-PNR in Figure 6b). Unfortunately, the spectrum B-FP-PNR is generally quite weak and it is hard to reveal reliably all the weak bands of the NR skeleton. Nevertheless, besides the dominant features of fingerprints, we detected several characteristics of the NR skeleton in the B-FP-PNR spectrum.

In summary, the spectra of the fingerprints (both in the absence and in the presence of PNR) on the brass plate exhibit clearly characteristic bands corresponding to the molecular composition of the fingerprints. Furthermore, in the spectrum of the PNR film, the characteristic bands of the NR skeleton are evidently supplemented with a new feature corresponding to the polymerization reaction. Finally, the presence of a PNR film can be detected in the case of fingerprints on the brass surface covered by PNR, but the PNR characteristics are weak, suggesting the pretty weaker adherence of PNR on fingerprint areas compared to the strong adherence of PNR on the original brass surface.

The spectroscopic experiments revealed that (i) adhesion of the polymeric film on the material with the deposited fingerprint is important for the application of electrochemical visualization, (ii) the type of substrate (e.g., brass, steel, platinum) requires careful selection of spectroscopic measurements, and (iii) knowledge of the chemical composition of fingerprints and their affinity to the substrate can help to determine the type of polymeric film and imply its deposition. The combination of spectroscopic and electrochemical approaches is a promising tool, which can be efficiently used in this field. Nevertheless, to clarify the adherence of polymeric films and fingerprints on various supporting materials and to follow clearly the fingerprints’ contrast, micro-spectroscopic tools (infrared microscopy/imaging and Raman micro-spectroscopy) can be valuable.

#### 3.4.2. Electrochemical Characterization

Electrochemical characterization of the properties of the brass substrate was performed by the CV method in KNO_3_ solution in the presence of [Fe (CN)_6_]^4−^/[Fe (CN)_6_]^3−^ ions to study the redox properties and electrochemical activity of the given surface [36,37].

The results of electrochemical characterization in the presence of a redox marker are presented in Supplementary Materials (Figure S3). All records show only a broad cathodic peak at approximately the same potential values, indicating that this system is not reversible.

In summary, brass in the presence of a redox marker, with or without a fingerprint, does not show much different properties, as the individual records differed only slightly during the experiments. However, the PNR film slows down the oxidation of the brass substrate in the presence of a redox marker.

### 3.5. Fingerprint Visualization on Fired Cartridges (from the Shooting Range to the Laboratory)

The final purpose was to test the applicability of the proposed approach on real samples, namely fired brass cartridges, and to compare the obtained experimental finding with the ones known from the literature [9,22,38]. A number of studies confirm that the visualization of fingerprints on fired cartridges presents a difficult task, mainly due to damage of the deposited fingerprints [9,13,15]. One of the initial tasks was to find the correct placement of cartridges in the electrochemical cell. Two procedures were applied to carry out electrochemical visualization.

The cartridge was cleaned, and then a fingerprint was applied and visualized. The second procedure consisted of cleaning the cartridge, as previously described in Section 2.3, “Experimental Procedures,” but then no new fingerprint was applied.

The aim of this experiment was to visualize the original fingerprint of the shooter, which was printed on the cartridge in the past before loading the weapon and firing. Despite the partial degradation of the fingerprints due to the aging of the sample, the voltammogram of the PNR application recorded for the first and the second visualization method was identical (not shown here). Based on these results, it can be concluded that the electrochemical visualization of a fingerprint by means of PNR deposition is not affected by either the age or the quality of the applied fingerprint on the surface of the cartridge.

**Figure 7:**
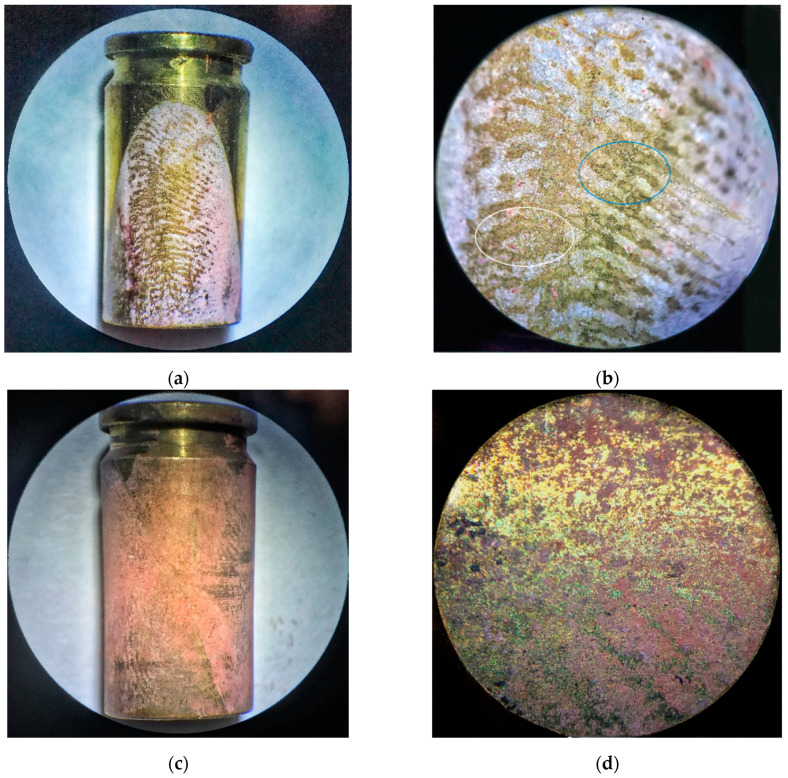
Visualized fingerprints on fired brass cartridges. Images were taken with a binocular magnifier: (**a**,**c**) 10× magnification and (**b**,**d**) 30× magnification. Comparison of two different fingerprints: (**a**,**b**) relatively quality fingerprint with marked details (cross (marked in blue) and fork (marked in white)) and (**c**,**d**) partial (poor-quality) print; both prints applied before firing (unknown shooters).

Figure 7 presents the comparison of images obtained using a binocular magnifier of visualized fingerprints left by two unknown shooters on the brass cartridges. It is possible to observe the difference in their quality, comparing Figure 7a–d. However, visualization of fingerprints located on the cartridge several months after the shot using the PNR film in Figure 7a,b allows observing and identifying the “markers” (details of second level). Both “crossing” (the blue ellipse) and “bicurcation” (the white ellipse) features can be easily observed (Figure 7b). This result can be considered successful [23] and proves the suitability of the technique used.

## 4. Conclusions

Currently, there is no universal method that can be used on all surfaces. The known visualization techniques (cyanoacrylate vapors, etc.) are quite effective on various kinds of paper or plastic surfaces. We proposed a visualization method suitable for brass surfaces that are materials of cartridges.

Reduction in the acidity of the supporting electrolyte, application of a narrow potential window at 6 cycles, and 0.002 M NR concentration leads to an optimal thickness of the polymerized PNR film and subsequently to qualitative visibility of the stored fingerprint. The fingerprint is clearly visible without further modification, thanks to the red color of the polymer film.

Spectroscopic characterization shows the presence in the fingerprint spectra of characteristic bands, indicating their molecular composition. The spectral response is also able to detect the presence of PNR spectral bands, indicating a polymerization reaction. The infrared spectra confirm a much weaker adhesion of PNR to the fingerprint area compared to the strong adhesion to the brass surface itself. The combination of spectroscopic and electrochemical approaches is a promising tool, which can be efficiently used in the forensic field in terms of fingerprint identification.

## Figures and Tables

**Table 1:** Comparing infrared spectra of powered neutral red (NR) and the PNR film deposited on the brass plate (brass-PNR).

Common Bands for Powdered NR and Brass-PNR (cm^−1^)	Assignments of Observed FTIR Bands
3325	N–H stretching vibrations in phenyleneamine fragments C–NH–C
3220	N–H stretching vibrations in C=NH groups
2919, 2858/2850, 2807/2803 ^a^	C–H stretching vibrations in–N(CH_3_)_2_
below 2800 ^b, c^ mainly 2735, 2658	amine hydrochloride
1600 ^b–d^ with two side peaks at 1652 and 1632	N–H deformation vibration of amines and/or –C=N stretching vibrations in heteroaromatic rings or further Ar–C=N– (in-plane) vibrations
1485 ^b, c^	Ring C–C stretching modes (vibrations of the quinonoid ring)
1435 ^b^	Aromatic in-plane stretching mode and bending vibrations of CH_3_ groups
1357, 1308, 1252 ^c, d^	Coupled C–N stretching vibrations
1173, 1162, 1145 ^b^	C–H in-plane bending vibrations of the aromatic rings
1035, 1010 ^c^	C–H bending vibrations of the aromatic rings
920, 878, 830, 810, 733, 717 ^b–d^	1,2,4-trisubstituted benzenes and 1,2,4,5-tetrasubstituted benzenes C–H out of plane (Oop) deformation and ring Oop deformation vibrations

^a^ The pairs of (slightly shifted) values separated by/corresponding to PNR/NR, respectively. ^b^ [32]. ^c^ [33]. ^d^ [34].

## Data Availability

Not applicable.

## Supplementary Materials

The following are available online at https://www.mdpi.com/article/10.3390/polym13193220/s1: Figure S1. Images of visualized fingerprints on brass plates (taken by mobile phone); Figure S2. Visualized fingerprint on brass. Images were taken with a Nikon stereoscopic microscope; Figure S3. Comparison of four measurements on brass at a scan rate of 10 mV∙s^−1^.
