# Supplementary material for: Optimization of Electrochemical Visualization of Latent Fingerprints with Poly(Neutral Red) on Brass Surfaces
Source: Polymers (Basel). 2021 Sep 23;13(19):3220. doi: 10.3390/polym13193220 (PMC8512869; doi:10.3390/polym13193220)
Supplement: Supplementary file 1 [file polymers-13-03220-s001.zip › polymers-1373482-supplementary.pdf]

Supplementary Materials

Visual characterization

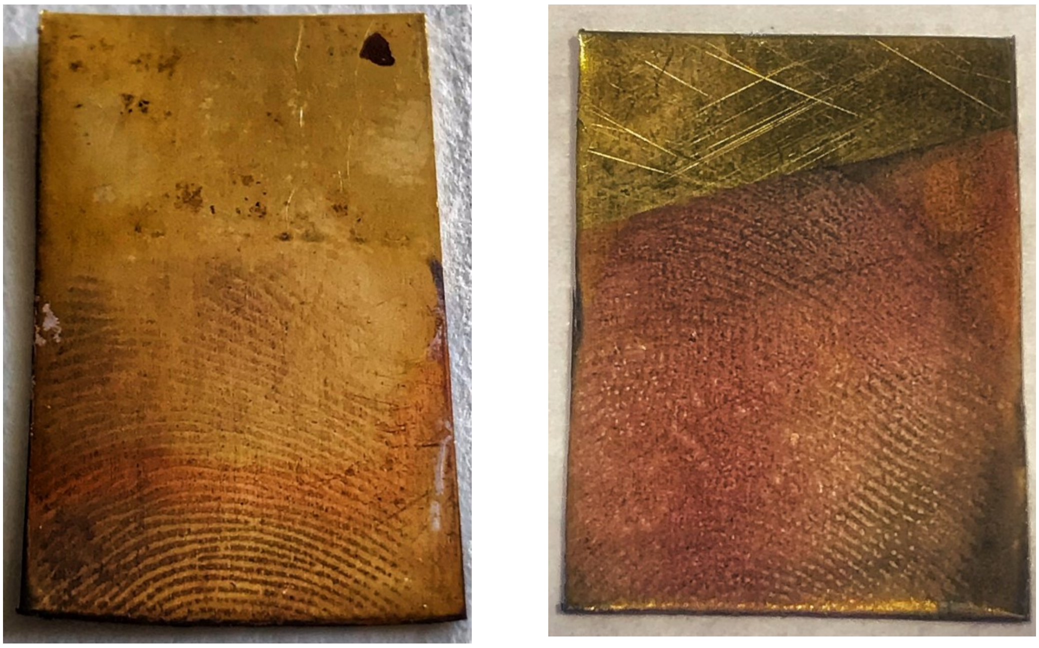

(**a**) (**b**)

Figure S1. Images of visualized fingerprints on brass plates (taken by mobile phone).

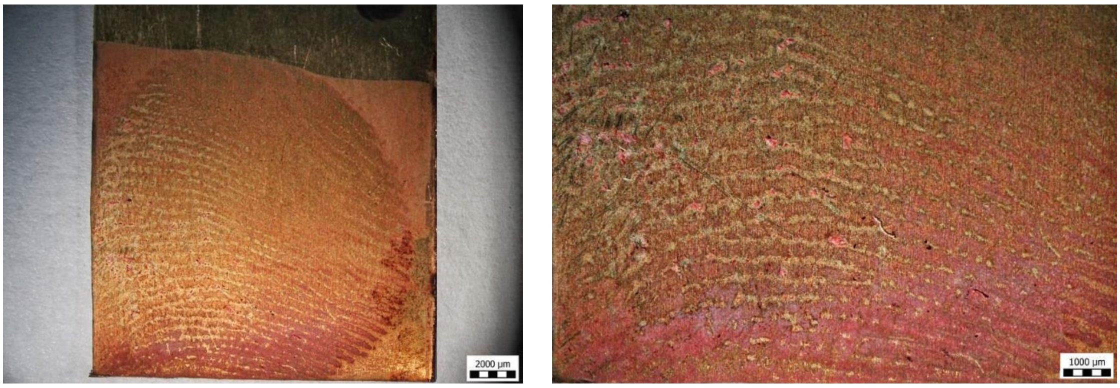

(**a**) (**b**)

Figure S2. Visualized fingerprint on brass. Images were taken with a Nikon stereoscopic microscope.

Electrochemical characterization

The electrochemical characterization aimed at comparing the redox properties of the metal surfaces themselves prior and after the application of the fingerprint or the polymer film PNR.

Electrochemical characterization of the properties of the brass substrate was performed by the CV method in the KNO_3 solution in the presence of $[Fe(CN)_6]^{4-}/[Fe(CN)_6]^{3-}$ ions to study the redox properties and electrochemical activity of the given surface [36,37]. This cation system is used to investigate the reversibility of the working electrodes. This method is based on a one-electron oxidation-reduction reaction of Fe^{2+}/Fe^{3+} ions on a given electrode surface [36]. In an ideal system of suitable conductive material where electron transfer occurs, two peaks should be recorded in the voltammogram, anodic (oxidative) and cathodic (reducing). In the case of a reversible system, the potential difference between the two peaks should be of 57 mV [37]. The height of both peaks should also be the same in absolute current values. After dividing the currents of the anodic and cathodic peaks, the value should be equal to one [36,37].

However, the current ratio value for both peaks can be greatly influenced by the chemical reactions accompanying redox processes. If the system is reversible, reduction of Fe^{3+} will be accompanied by re-oxidation of Fe^{2+} and vice versa. However, if electron transfer is prevented and the material is not sufficiently conductive, the system becomes irreversible and the peaks are no longer the same, or one of the peaks may not be recorded at all [36]. Another parameter that proves the reversibility of the system is the ratio of current and scan speed. If the system behaves reversibly, the absolute value of the current increases linearly with the square root of the scan speed [37].

Four different samples were characterized using cyclic voltamograms:

1st sample - pure brass,

2nd sample - brass with fingerprint on both sides,

3rd sample - brass coated with PNR on both sides,

4th sample - brass with fingerprint visualized by the PNR film on both sides (Fig. S3).

The CV voltamograms were very similar regardless the type of the tested samples. It is possible to confirm that a position and intensity of cathodic peak observed in the range from 1 to -80 mV can be resulted by the effect of chemical composition of fingerprint on redox properties of both brass substrate and deposited PNR film (Fig.S3).

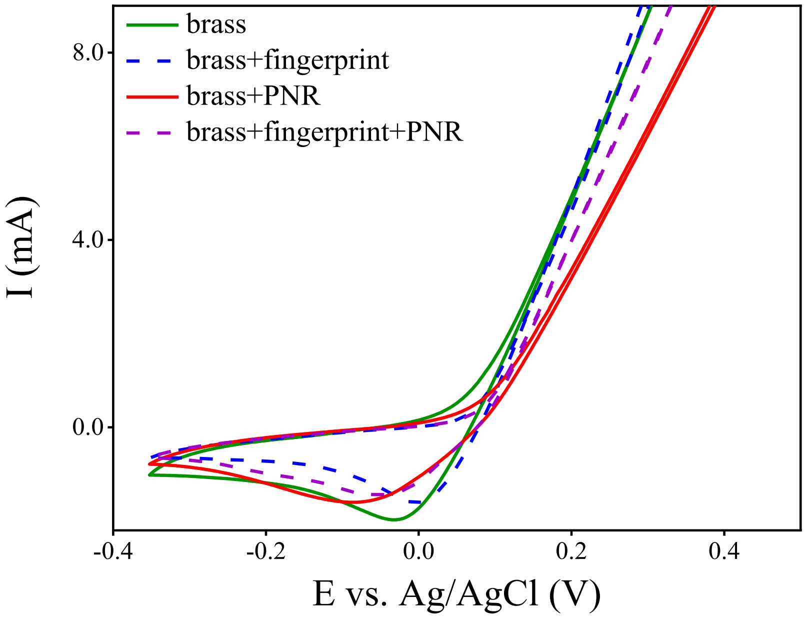

Figure S3. Comparison of four measurements on brass at scan rate 10 mV·s^{-1}.
